# Acute death caused by invasive aspergillosis after living-donor liver transplantation despite good graft function: a case report

**DOI:** 10.1186/s40792-021-01203-w

**Published:** 2021-05-12

**Authors:** Takahiro Tomiyama, Takashi Motomura, Norifumi Iseda, Akinari Morinaga, Tomonari Shimagaki, Takeshi Kurihara, Huanlin Wang, Takeo Toshima, Yoshihiro Nagao, Shinji Itoh, Noboru Harada, Tomoharu Yoshizumi, Masaki Mori

**Affiliations:** Department of Surgery and Sciences, Graduate School of Medical Sciences, Kyushu University Hospital, Kyushu University, 3 Chome-1-1 Maidashi, Higashiku, Fukuoka, Fukuoka 812-8582 Japan

**Keywords:** Invasive aspergillosis, Liver transplantation, Antifungal treatment

## Abstract

**Background:**

Invasive aspergillosis (IA) is one of the most serious causes of death after liver transplantation (LT). IA is the second most common fungal infection, and its mortality rate exceeds 80%.

**Case presentation:**

A 67-year-old man presented to our hospital because of fulminant hepatitis caused by hepatitis B virus. Candidiasis was detected in his sputum, and micafungin had already been administered. Living-donor LT was performed using a right lobe graft donated from his daughter with no intraoperative complications. Although he appeared to have good graft function, his oxygenation was inadequate, and a chest radiograph showed many invasive shadows on postoperative day 1. A computed tomography scan also showed many invasive shadows with the halo sign. A blood examination revealed positivity for *Aspergillus* antigen, and *Aspergillus* species were detected in his sputum. IA was diagnosed. The antifungal therapy was soon modified to amphotericin B combined with caspofungin. Despite good graft blood flow through the portal vein and hepatic artery and good graft function, the patient died of IA on postoperative day 3. The median time from LT to IA among reports published to date ranges from 18 to 25 days.

**Conclusions:**

The present report describes the first case of very early onset of IA after LT.

## Background

Fungal infection is one of the most serious causes of death after liver transplantation (LT) [1]. In particular, invasive aspergillosis (IA) is the second most common fungal infection, and its mortality rate exceeds 80% [2, 3]. The mortality rate of untreated aspergillosis has been found to approach 100% [4]. Considering the high mortality rate of IA, identification of risk factors and effective antifungal prophylactic agents is urgently needed.

The onset of IA reportedly occurs at a median of 18 to 25 days after LT [5, 6] The incidence of IA within 1 day after LT has rarely been reported [7]. We herein describe a patient in whom the onset of IA occurred 1 day after LT.

## Case presentation

A 67-year-old man visited his family physician because of anorexia and fatigue. He was diagnosed with heatstroke and treated by drip infusion. However, his symptoms did not improve, and he revisited the clinic on the third day after the initial presentation. Jaundice was found at that time, and he was referred to the general hospital for further evaluation. His laboratory data revealed acute liver failure as indicated by an aspartate aminotransferase concentration of 11,218 U/L, alanine aminotransferase concentration of 9974 U/L, lactate dehydrogenase concentration of 10,070 U/L, and prothrombin percentage activity of 12%. Although he had been negative for hepatitis B virus surface antigen in 2009, the current laboratory data showed positivity for this antigen, suggesting that the cause of the acute liver failure was acute hepatitis B virus infection. He also exhibited hepatic coma with an elevated ammonia concentration of 480 μg/dL and was transferred to the advanced treatment hospital on the fourth day, where lamivudine was administered and steroid pulse therapy (1000 mg of methylprednisolone for 3 days, followed by 500 mg for three days and 250 mg for 3 days), plasma exchange, and high-flow continuous hemodiafiltration were performed. Moreover, *Staphylococcus hominis* and *Candida* species were detected from his blood culture and sputum, respectively. Vancomycin, meropenem, and fluconazole (FLCZ) were administered. His blood culture was confirmed to be negative on the sixth day. However, his liver function worsened; therefore, he was referred to our hospital for LT on the 12th day.

**Fig. 1:**
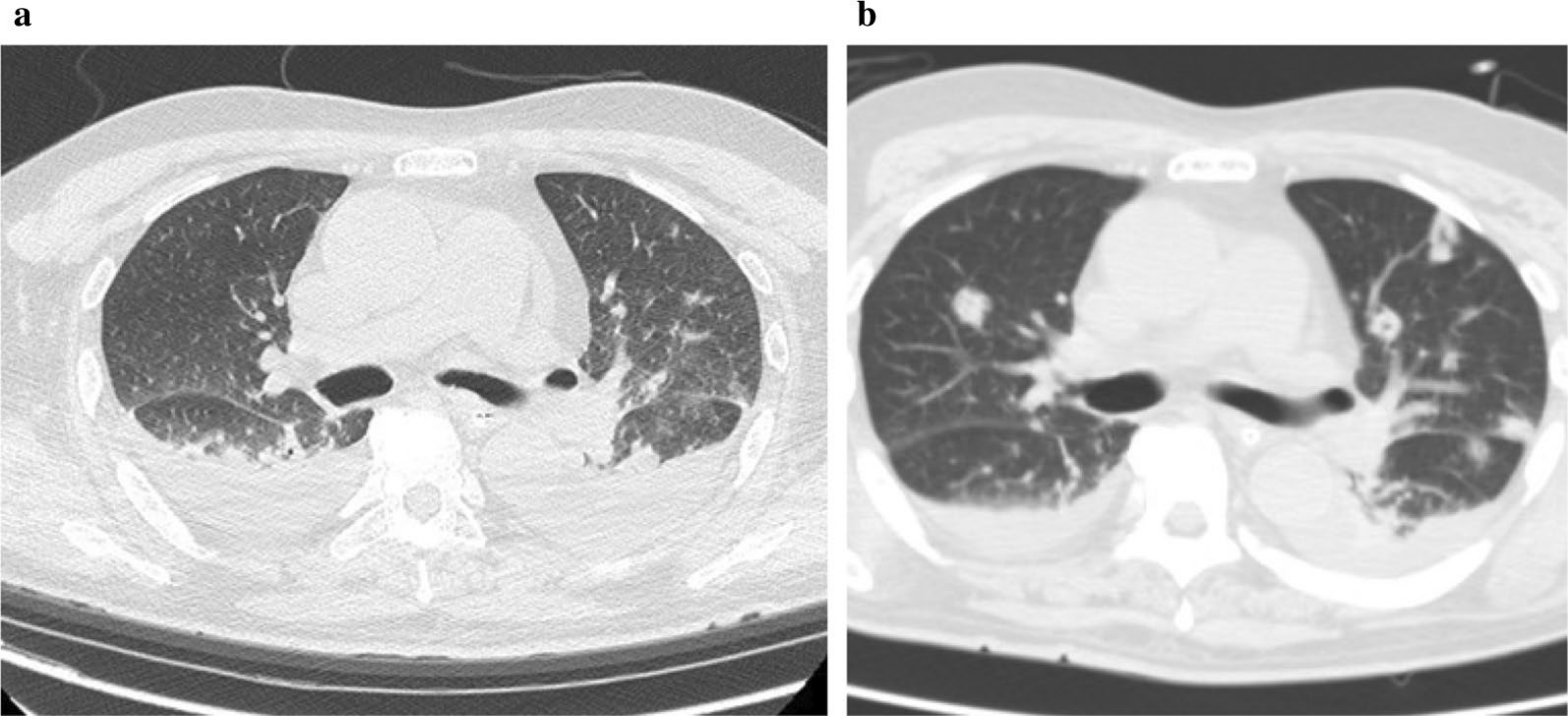
**a** Computed tomography scan performed 9 days before living-donor liver transplantation. Only a small amount of pleural effusion was present. **b** Computed tomography scan performed 3 days before living-donor liver transplantation. Some small nodular shadows were observed

Upon transfer to our hospital, the patient was intubated and his Glasgow Coma Scale score was E3VTM5. He had no fever. He was admitted to the intensive care unit (ICU). Blood examination indicated acute liver failure (total bilirubin, 7.8 mg/dL; aspartate aminotransferase, 20 U/L; alanine aminotransferase, 31 U/L; lactate dehydrogenase, 264 U/L; blood urea nitrogen, 2 mg/dL; ammonia, 178 μg/dL; prothrombin percentage activity, 26%; and prothrombin time–international normalized ratio, 2.74). His Model for End-Stage Liver Disease (MELD) score was 39. Although no nodules had been present on the first computed tomography (CT) scan (Fig. 1a), multiple small nodular shadows were found in both lungs on the next CT scan (Fig. 1b). Septic emboli due to bacterial infection were suspected, and vancomycin, meropenem, and FLCZ were continued.

On the 14th day, living-donor LT was performed using a right lobe graft donated from his daughter with no intraoperative complications. The operation time was 12 h, and the estimated blood loss was 8805 g.

**Fig. 2:**
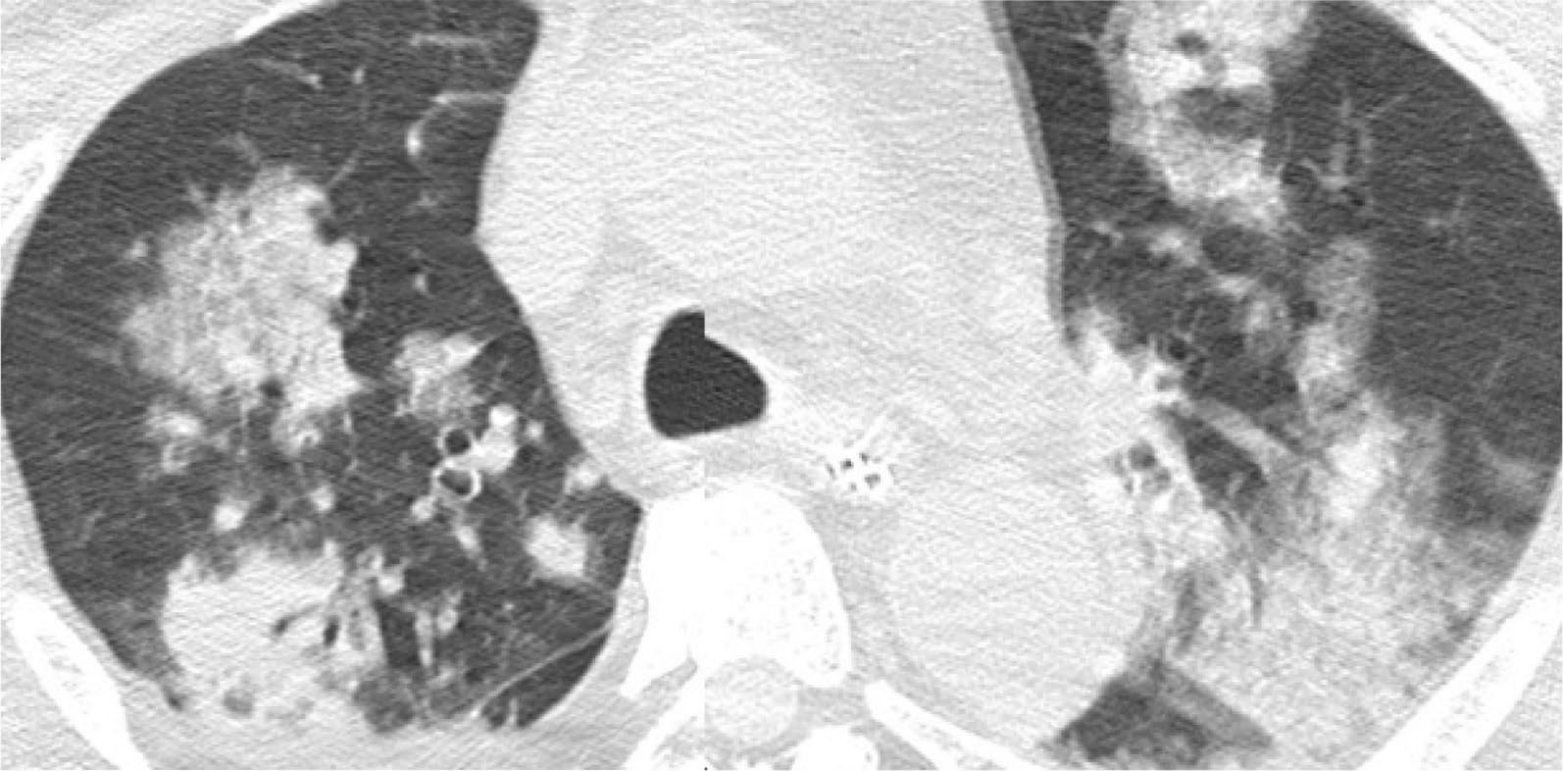
Computed tomography scan performed on postoperative day 1. Nodular shadows with the halo sign were observed

On postoperative day (POD) 1, sedation was discontinued but the patient did not regain clear consciousness. In addition, the invasive shadow on the chest radiograph had obviously grown. CT showed that the nodular shadows had increased in both size and number, and the halo sign was present (Fig. 2). The possibility of a fungal infection was considered, including *Candida* and *Aspergillus* species, and the antifungal agent was changed from FLCZ to amphotericin B (AMPH-B) and caspofungin (CPFG).

On POD 2, the shadow on the chest radiograph remained almost unchanged, but the patient’s consciousness was worsening. A blood test showed positive results for *Aspergillus* antigen and negativity for *Candida* mannan antigen, *Cryptococcus* antigen, T-SPOT.TB, interferon gamma release assay and *Cytomegalovirus* pp65 antigen. Moreover, *Aspergillus* species were detected in his sputum, and IA was diagnosed. The graft blood flow through the portal vein and hepatic artery and the graft function were good. Although methylprednisolone was discontinued and AMPH-B and CPFG were continued for treatment of the *Aspergillus* infection, the patient died of respiratory failure due to IA on POD 3.

## Discussion

Fungal infections are seriously life-threatening conditions for liver LT recipients. The reported occurrence of serious invasive fungal infections in patients undergoing LT ranges from 7 to 42% [8]. In particular, IA has been reported in 1.5% to 6.5% of patients undergoing LT [9]. Aspergillosis is reportedly the second most common fungal infection following candidiasis in LT recipients [10].

**Fig. 3:**
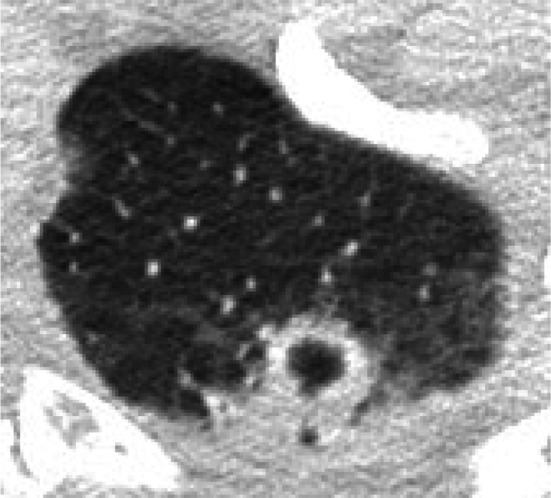
Another computed tomography scan performed 2 days before living-donor liver transplantation. A small nodular-shaped halo sign was observed

Diagnosis for IA needs a comprehensive and rigorous workup, including a combination of histopathology, microbiology, serology and imaging data [11]. According to the European Organization and Research and Treatment of Cancer/Invasive Fungal Infections Cooperative Group and the National Institute of Allergy and Infectious Disease Mycoses Study Group (EORTC/MSG) criteria [12], our case could be classified as nothing more than possible IA before LT (Table 1), since no fungal elements indicating a mold was detected from his sputum before LT. Sensitivities of traditional diagnostic approaches including staining with Gomori’s methenamine silver or periodic acid–Schiff (PAS) stains and fungal cultures of clinical specimen were reported to vary as 20–70% [11]. On the other hand, several other useful examinations are available to detect or suspect IA. First, the serum or broncho-alveolar lavage galactomannan (GM) assay is useful. The serum GM has shown good diagnostic performance with sensitivity of a 78% and specificity of 85% in patients with an *Aspergillus*-positive culture of a normally sterile specimen or with clinical features suspicious for IA [1]. The BAL GM is more useful than serum GM. Lahmer et al. [13] reported that sensitivity of 90% and specificity of 85% was found for BAL GM. Second, CT scan is also useful. On a CT scan imaging, IA is characterized by the appearance of the halo sign. Qin et al. [14] reported that 80% of patients with IA showed the halo sign within 1 week after symptom onset. In fact, upon reviewing our patient’s CT scan, a small nodular-shaped halo sign was observed (Fig. 3). In our case, antifungal treatment with FLCZ had already been administered for candidiasis detected in the patient’s sputum before LT. Therefore, when the last CT scan before LT showed multiple small nodules in the lung fields, fungal balls were not suspected. However, consideration should have been given to the drug sensitivity; FLCZ is not sensitive for *Aspergillus* species. Despite modification of the antifungal therapy to AMPH-B combined with CPFG (the standard and most effective regimen for IA) immediately upon diagnosis of IA, and despite good liver graft function, the patient died on POD 3. In the current case, IA after LT was lethal and too difficult to recover. Detection or suspicion of IA as early as possible is more important that than initially knowing how to treat. If the GM assay had been performed or halo sign had been detected before LDLT in our case, he might have been diagnosed as probable IA and been treated for IA earlier.

**Table 1:** Fungal Infectious Cooperative Group and the National Institute of Allergy and Infectious Diseases Mycoses Study Group (EORTC/MSG) criteria for IA [12]       (The items which met the current case before LT are described in bold)

**Proven IA**
Microscopic analysis on sterile material: a specimen obtained by needle aspiration or sterile biopsy in which hyphae are seen accompanied by evidence of tissue damage or culture on sterile material for Aspergillus becomes positive
**Probable IA** (requires at least one item for each factor, but mycological criteria are absent for proven IA criteria)
[Host factors]
Recent history of neutropenia (< 500 neutrophils/mm^3^ for > 10 days)
Recipient of an allogeneic stem cell transplant
Prolonged use of corticosteroids at a dose of 0.3 mg/kg/day of prednisone equivalent for > 3 weeks
Treatment with other recognized T cell immunosuppressants during the past 90 days (such as TNF-α blockers, specific monoclonal antibody, or **nucleoside analogues**)
Inherited severe immunodeficiency (such as chronic granulomatous disease or severe combined immunodeficiency)
[Clinical criteria]
Lower respiratory tract fungal disease
The presence of one of the following three signs on CT
**Dense, well-circumscribed lesion(s) with or without a halo sign** Air-crescent sign **Cavity**
[Mycological criteria]
Mold in sputum, BAL, fluid, bronchial brush, indicated by 1 of the following
Presence of fungal elements indicating a mold
Recovery of Aspergillus by culture or indirect tests (detection of antigen or cell wall constituents)
Galactomannan antigen detected in serum or BAL fluid
β-d Glucan detected in serum

*IA* invasive pulmonary aspergillosis, *LT* liver transplantation, *TNF-α* tumor necrosis factor-alpha, *CT* computed tomography, *BAL* broncho-alveolar lavage

Voriconazole is the first-line treatment for IA based on the international guidelines [15, 16] since its better results and fewer side effects had been reported in 2002 than those of the initial therapy with amphotericin B [17]. However, there still remain several problems in treatment for IA with voriconazole in LT. First, co-administration of triazoles, including particularly voriconazole and posaconazole with immunosuppressive drugs, such as tacrolimus and sirolimus possibly encompasses a drug–drug interaction. These antifungal drugs inhibit the CYP3A4 activity so that they would fluctuate the concentration of the calcineurin inhibitor [18]. Second, potential liver toxicity associated with triazole should be concerning, especially for the recipients in the early period of LT [11]. The onset of IA in the current case is also very early after LT. Hence, voriconazole was considered to be avoided and AMPH-B was used, which is a primary alternative therapy for IA [11]. Abe, et al. reported a case with IA which occurred on 2POD after LDLT and they achieved complete cure of IA with voriconazole administration and lung resection, but the patients died of liver failure. They did not mention the cause of liver failure, but the possibility that voriconazole might have played some role in causing liver failure cannot be denied. On the other hand, isavuconazole has some potential to reduce the load on these problems. Isavuconazole has been indicated to have equal efficacy compared to voriconazole [19] and has been shown to have fewer adverse effect such as hepatic and neurological toxicities, and drug–drug interactions, including tacrolimus and sirolimus [20]. However, this promising drug has not been approved yet for treatment for IA, at least in Japan. Third, caution should be paid to voriconazole resistance. A previous study reported that 16.2% of Aspergillus fumigatus, the major species of Aspergillus, were resistant to voriconazole and that other less-prevalent Aspergillus species also showed occasional resistance to voriconazole [21]. However, some institutes, including our hospital, require several weeks or months to conduct a susceptibility test [21]. Since immediate drug intervention is needed for IA, this time discrepancy makes the treatment for IA difficult and should be solved urgently.

In Japan, the frequency of IA after LDLT was previously reported to be 0.7–1.0% [3, 21], which is less than in other countries. One of the possible reasons for fewer IA cases after LT in Japan, is because LDLT is dominant instead of deceased donor liver transplantation due to the extreme shortage of deceased donor. The risk factors for IA in patients undergoing LT are acute liver failure, renal replacement therapy, re-transplantation, reoperation, and preoperative steroid administration [3, 10, 22]. LDLT could have reduced the exposure time to these risks, which were some standard therapies for acute liver failure.

Balogh et al [22]. reported that voriconazole prophylaxis was effective in preventing IA in recipients with a MELD score of > 25 or who met at least two of the following criteria: ICU admission > 24 h before LT, hemodynamic instability requiring the use of inotropic support, renal disease requiring chronic renal replacement therapy, fulminant hepatic failure, combined liver and kidney transplantation, and pre-LT respiratory failure requiring mechanical ventilation. AMPH-B prophylaxis is also effective in preventing IA in high-risk patients [23]. Our patient’s MELD score was 39 (> 25), and he had three of the above-mentioned risk factors (pre-LT ICU admission, acute liver failure, and pre-LT respiratory failure requiring mechanical ventilation). Prophylactic treatment might have been needed in our high-risk case.

In our institution, all candidate recipients for LT are routinely checked for bacterial culture positivity, *Candida* antigen, *Cytomegalovirus* antibody, Epstein–Barr virus antibody, human immunodeficiency virus antigen, and human T cell leukemia virus type 1 antigen, but not for *Aspergillus* antigen. No one can know the development of IA on first days after LT. However, if IA could be suspected before LT, an additional strategy could be implemented, including prophylactic anti-aspergillosis treatment. Taking into account the limited number of deceased organ donors and the risk for living donor, we even have to consider the discontinuation of LT because of its high mortality.

## Conclusion

This is the first detailed report of very early onset of IA after LT. More attention should be given to the possibility of IA before LT. A new strategy including therapeutic drugs and diagnostic tools are needed.

## Data Availability

Not applicable.

## Abbreviations

AMPH-B: Amphotericin B
CPFG: Caspofungin
CT: Computed tomography
EORTC/MSG: European Organization and Research and Treatment of Cancer/Invasive Fungal Infections Cooperative Group and the National Institute of Allergy and Infectious Disease Mycoses Study Group
IA: Invasive aspergillosis
ICU: Intensive care unit
FLCZ: Fluconazole
LT: Liver transplantation
MELD: Model for End-Stage Liver Disease
POD: Postoperative day
